# Molecular Design of Encapsulin Protein Nanoparticles to Display Rotavirus Antigens for Enhancing Immunogenicity

**DOI:** 10.3390/vaccines12091020

**Published:** 2024-09-06

**Authors:** Hyun-Gyo Jung, Seonghun Jeong, Min-Ji Kang, Ingi Hong, Young-Shin Park, Eunbyeol Ko, Jae-Ouk Kim, Deog-Young Choi

**Affiliations:** 1 InThera, Inc., Seoul 05836, Republic of Korea; lucyplusk@inthera.co.kr; 2 Molecular Immunology, Science Unit, International Vaccine Institute, Seoul 08826, Republic of Korea; seonghun.jeong@ivi.int (S.J.); minji.kang@ivi.int (M.-J.K.); ingi.hong@ivi.int (I.H.); youngshin.park@ivi.int (Y.-S.P.); eunbyeol.ko@ivi.int (E.K.)

**Keywords:** encapsulin, nanoparticle, rotavirus, vaccine, VP8, immunization, neutralizing response

## Abstract

Rotavirus considerably threatens global health, particularly for children <5 years. Current, licensed oral attenuated vaccine formulations have limitations including insufficient efficacy in children in low- and middle-income countries, warranting urgent development of novel vaccines with improved efficacy and safety profiles. Herein, we present a novel approach utilizing an encapsulin (ENC) nanoparticle (NP)-based non-replicating rotavirus vaccine. ENC, originating from bacteria, offers a self-assembling scaffold that displays rotavirus VP8* antigens on its surface. To enhance the correct folding and soluble expression of monomeric antigens and their subsequent assembly into NP, we adopted an RNA-interacting domain (RID) of mammalian transfer RNA synthetase as an expression tag fused to the N-terminus of the ENC-VP8* fusion protein. Using the RID-ENC-VP8* tripartite modular design, insertion of linkers of appropriate length and sequence and the universal T cell epitope P2 remarkably improved the production yield and immunogenicity. Cleavage of the RID rendered a homogenous assembly of ENC-P2-VP8* into protein NPs. Immunization with ENC-P2-VP8* induced markedly higher levels of VP8*-specific antibodies and virus neutralization titers in mice than those induced by P2-VP8* without ENC. Altogether, these results highlight the potential of the designed ENC NP-based rotavirus vaccine as an effective strategy against rotavirus disease to address global health challenges.

## 1. Introduction

Rotavirus is a non-enveloped virus with double-stranded RNA and the leading cause of acute diarrhea, presenting high mortality and morbidity rates among young children [1]. Live attenuated oral rotavirus vaccines have proven to be generally safe and effective in preventing severe rotavirus diarrhea. However, concerns persist regarding rare but severe adverse events such as intussusception, along with lower vaccine effectiveness in low- and middle-income countries [2]. Many rotavirus vaccines have been developed for parenteral administration as alternatives to oral rotavirus vaccines [3].

Among them, self-assembling nanoparticle (NP)-based vaccines offer multiple advantages: (1) enhanced adaptive immune response and providing long-lasting protection by mimicking the size and structure of pathogens, and (2) protecting vaccine components from degradation, thus increasing stability [4,5]. Therefore, the NP-based non-replicating rotavirus vaccine (RV) is an innovative approach aimed at enhancing the efficacy and safety for efficient prevention of viral infection.

However, the fusion of antigenic proteins to particle scaffolds often disrupts correct folding, leading to the formation of misfolded insoluble precipitates, a phenomenon influenced by the size and structure of the fusion partner [6]. Previous studies have reported this challenge with rotavirus VP8, where fusion with Lumazine synthase resulted in the expression of inclusion bodies [7].

Herein, we engineered chimeric NPs (cNPs) displaying rotavirus VP8* antigens on the surface of encapsulin (ENC) as a scaffold for assembly. ENC, derived from bacteria, is known for its efficient expression and ability to assemble into highly repetitive structures within bacterial cells [8,9]. However, fusing an antigen for display on the surface of ENC often renders the fusion chimeric construct insoluble owing to the kinetic complexities involved in the folding and assembly of the chimeric protein. ENC-VP8* fusion protein is expressed in an insoluble form in *Escherichia coli*. To overcome this challenge and ensure correct folding and soluble expression, we employed an RNA-interaction-mediated folding system where the RNA-interacting domain (RID) was derived from mammalian transfer RNA (tRNA) synthetase as an expression tag fused to the N-terminus of ENC-VP8*. Thus, a tripartite construct, RID-ENC-VP8*, was designed: RID as a tRNA-binding tag, ENC as a scaffold for NP assembly, and VP8* as the target antigen. This chimeric design enabled the predominant expression of the protein in soluble form.

A significantly improved production yield and immunogenicity were achieved through an exploratory approach involving the insertion of appropriate linkers. Using a simple two-step purification process, we obtained purified particles with >90% purity (including clarification step). Physicochemical analyses, including dynamic light scattering (DLS) and transmission electron microscopy (TEM), confirmed the homogeneous assembly of ENC-P2-VP8* into protein NPs (PNPs). Immunization with ENC-P2-VP8* NP greatly enhanced VP8*-specific antibody responses and rotavirus neutralization titers in mice compared with those induced using monomeric P2-VP8*.

Based on the aforementioned findings, it can be deduced that the designed ENC NP-based rotavirus vaccines offer a promising strategy against rotaviruses. Harnessed via RNA-assisted folding [10,11], these self-assembled NP vaccines demonstrated stable, high-yield expression in bacteria, ensuring efficient production.

## 2. Materials and Methods

### 2.1. Plasmid DNA (pDNA)

Rotavirus VP8*, spanning regions 65 to 223 of the VP8 protein and known for its RNA-binding domain of Wa strains with protein sequences from the National Center for Biotechnology Information (NCBI; GenBank accession numbers: FJ423116 [Wa], PDB: 1KQR), was synthesized via gene optimization for *E. coli*. The protein sequence of ENC (1–264 region) from *Thermotoga maritima* was retrieved from NCBI (GenBank accession number: CP011107.1; PDB: 3DKT). RID was derived from the N-terminal domain of human LysRS [12]. Previously, RID has been shown to interact with tRNAs de novo and facilitate protein folding and soluble expression [13,14]. Expression tags, including the RID, 6-histidine tag, and tobacco etch virus (TEV) protease cleavage site, were excised from pGE-hRID(3) using the restriction enzymes NdeI and BamHI [15]. Digested fragments were inserted into a vector (Novagen, Madison, WI, USA) to generate pET9a-RID-6xHIS-TEV-ENC-Linker-VP8*. Vectors containing only the ENC sequence (pET9a-ENC) and the ENC sequence with the target antigen VP8* (pET9a-ENC-VP8*) were generated to compare the expression of proteins in bacteria. A stop codon was inserted immediately after sequencing the recombinant protein.

pDNAs were utilized to study the effect of linkers and tetanus toxin T cell epitope P2 on protein expression and immunogenicity. Vectors encoding proteins in combination with different linkers, with or without P2, were cloned. Furthermore, vectors were generated to compare the effects of different linkers, with linker 1 (L1) and linker 2 (L2) at the C-terminus of ENC, respectively, in the presence of (GS)_3_ preceding ENC. As controls, two expression constructs were designed, one with a completely flexible linker L1, (G_3_S)_3_SGGS, and another linker L2 carrying the semi-flexible sequence EAAAK ([G_3_S]_2_EAAAKG_3_S), which reduced the interference between the domains [16,17]. This resulted in the formation of two vaccine constructs. One was a fusion of RV VP8* to ENC VP8* with L1, referred to as ENC-L1-VP8*. The second construct was a fusion of RV VP8* to ENC with L2, referred to as ENC-L2-VP8*. Previous studies suggested that the two versions of ENC-VP8* protein may differ in particle formation and immune responses. Additionally, it consists of the tetanus toxin T cell epitope P2 linked to VP8*, as previously described [18,19].

### 2.2. Production and Purification of Recombinant Protein pDNAs

Plasmids were transformed into *E. coli* BL21 (DE3) competent cells using heat shock to express the recombinant proteins. Kanamycin-resistant colonies were cultured until the optical density at 600 nm (OD_600_) of approximately 0.6 in 500 mL in a lysogeny broth medium containing 50 µg/mL kanamycin, at 37 °C with agitation (250 rpm). Thereafter, 5 mL of this culture was diluted to 1 L and grown until the OD_600_ reached approximately 0.6. At this point the culture was induced by adding 0.4 mM isopropyl-β-d-thiogalactopyranoside and incubated, followed by overnight incubation at 16 °C with agitation (250 rpm). Samples were harvested via centrifugation at 4000× *g* for 20 min at 4 °C.

### 2.3. Production of ENC-Based Nanoparticles (NPs)

#### 2.3.1. Cell Lysis and Initial Preparation

After centrifugation, the pellet was sonicated in 20 mL of lysis buffer consisting of 50 mM Tris–HCl, 150 mM NaCl, 5 mM imidazole (pH 7.9), 0.02% Tween-20, and 5% glycerol. After incubation on ice for 30 min, sonication was performed on the ice twice under conditions of 70% amplitude, 15 s/50 s pulse on/off, 2 min 30 s cycle, and 2 min rest using an ultrasonic processor (VCX500, SONICS vibra cell™, Newton, MA, USA). The cell lysate was clarified at 13,500 rpm for 10 min at 4°C. The supernatant was filtered through 0.45 µm and 0.22 µm syringe filters (Sartorius, Göttingen, Germany).

#### 2.3.2. Purification of ENC-Based NPs

The filtered supernatant of each lysate was subjected to immobilized metal affinity chromatography (IMAC) using a Ni-affinity Histrap™ HP column by ÄKTA^®^ Prime chromatography system (GE Healthcare, Stockholm, Sweden). The RID and 6× HIS tag at the N-terminus of ENC-based particles and P2-VP8* were removed via TEV protease treatment. The cleaved sample without 6× HIS was eluted during flow-through. Two different buffers were used for the chromatography. Buffer A contained 50 mM Tris-HCl (pH 8.0), 150 mM NaCl, 5% glycerol, 0.2% Tween-20, and 10 mM imidazole, and buffer B contained 50 mM Tris-HCl (pH 8.0), 150 mM NaCl, 5% glycerol, 0.2% Tween-20, and 1 M imidazole. These buffers were used in different proportions for the equilibration, washing, and elution of IMAC.

#### 2.3.3. Final Purification and Quality Control

To enhance the purity and remove endotoxins, purification was performed via multimodal chromatography using a HiTrap Q FF, 1 mL size (GE Healthcare Life Sciences, Little Chalfont, Buckinghamshire, UK), operating in flow-through mode. Both chromatographic steps were performed using an ÄKTA Purifier protein purification system controlled using the software Unicorn 5.20 (GE Healthcare, Stockholm, Sweden). For all chromatographic experiments, the flow rate was 1 mL/min, and fractions were collected in 2 mL aliquots unless stated otherwise. Further purification was performed in two chromatographic steps, followed by anion exchange chromatography using a HiTrap QFF and TMAE 1 mL membrane (Sartorius, Göttingen, Germany) in bind-and-elute mode. Two different buffers were used for chromatography. Buffer A contained 50 mM Tris-HCl (pH 7.5), 10 mM NaCl, 5% glycerol, and 0.2% Tween-20, and buffer B contained 50 mM Tris-HCl (pH 7.5), 1 M NaCl, 5% glycerol, and 0.2% Tween-20. These buffers were used in different proportions in equilibration, washing, and elution steps. The concentration of endotoxin-depleted particles was determined using a bicinchoninic acid assay (Thermo Fisher Scientific, København V, Denmark), and particles were stored at 4 °C. Protein analysis was performed using sodium dodecyl sulfate-polyacrylamide gel electrophoresis (SDS–PAGE).

### 2.4. Characterization of ENC-Based NPs with TEM

TEM was performed for the microscopic evaluation of the size and structure of the purified NPs. A drop of NPs (0.1 mg/mL, 5 μL) was placed on a formvar/carbon-coated TEM grid. The grid was accelerated with a glow discharge cleaning system (PELCO easiGlow™) to make it available before the staining. The grid was negatively stained with 2% uranyl acetate, dried, and examined using a Talos L120C Transmission electron microscope (FEI, Brno, Czech Republic) at an accelerating voltage of 120 kV. Particles were randomly captured in an image field, and their sizes were measured for characterization. The TEM results confirmed that the purified ENC-VP8* protein assembled into cNPs.

### 2.5. DLS Analysis of ENC-Based NPs

DLS was used to analyze the diameter distribution of the samples using a zeta potential and particle size analyzer, Zetasizer Nano (Malvern Panalytical, Malvern, Worcestershire, UK). Before each measurement, the quartz cuvette was incubated in the DLS instrument for 5 min to stabilize the sample temperature at 16 °C. Samples (1 mL) with 125–250 µg/mL total protein concentration were measured with 20 scans of 10 s each. The diameter distribution of the sample was measured using water as a solvent with an accumulation time of 200 s.

### 2.6. Mouse Immunizations with ENC-Based NPs

Six-week-old female BALB/c mice (Koatech, Pyeongtaek, Republic of Korea) were purchased and housed in the Animal Research Facility of the International Vaccine Institute (Seoul, Republic of Korea) under specific pathogen-free standard laboratory conditions. All animal experiments were approved by the IVI Institutional Animal Care and Use Committee (No. 2023-011). Vaccine antigens and alum hydroxide (Alhydrogel 2%) as an adjuvant were co-administered at low, mid, and high doses corresponding to 0.01 nmol (0.2 µg P2-VP8* and 0.5 µg ENC-P2-VP8*), 0.05 nmol (1 µg P2-VP8* and 2.6 µg ENC-P2-VP8*), 0.24 nmol (5 µg VP8*, 5 µg P2-VP8*, 12.5 µg ENC-VP8*, and 12.5 µg ENC-P2-VP8*), and 0.25 nmol (5.1 µg P2-VP8* and 13.1 µg ENC-P2-VP8*), respectively. Formulations were incubated at 25 °C for 1 h to facilitate antigen adsorption to the adjuvant. Mice were immunized intramuscularly thrice at 2-week intervals with 100 µL in phosphate-buffered saline (PBS) per vaccination, alternately administered to the quadriceps of both legs, with 50 µL per leg. To assess the humoral immune responses, mouse sera were collected 13 d after each immunization.

### 2.7. Detection of Binding Antibodies by Enzyme-Linked Immunosorbent Assay

Herein, 96-well immunoplates (Thermo Fisher Scientific, København V, Denmark) were coated with 200 ng/well recombinant rotavirus VP8* protein in 100 µL of PBS with 1% bovine serum albumin (BSA) and incubated overnight at 4 °C. Plates were washed thrice with washing buffer (PBS containing 0.05% Tween 20) and blocked with 1% BSA in PBS for 1 h at room temperature. Diluted sera and anti-rotavirus P [8]VP8 monoclonal antibody 16H7 as a positive control were added to wells and incubated at room temperature for 2 h. After washing four times with washing buffer to remove unbound antibodies, wells were incubated with appropriate dilutions of goat anti-mouse IgG, IgG1, and IgG2a horseradish peroxidase (HRP) for 1 h at room temperature. After five washes with washing buffer, 100 µL of 3,3′,5,5′-tetramethylbenzidine (Rockland, Limerick, PA, USA) substrate reagent was added. Upon color development, 50 µL of 0.5 N HCl (Sigma, Taufkirchen, Germany) was added to stop the reaction. Antibody titers were calculated as log2 of the reciprocal dilution, showing an optical density above the cutoff value, and measured using a microplate reader (Molecular Devices, San Jose, CA, USA). The optical density at 650 nm was subtracted from that at 450 nm.

### 2.8. Measurement of Neutralization Activity in Serum against Rotavirus

MA104 cells were seeded at 1.0 × 10^4^ cells/well in 96-well plates (Thermo Fisher Scientific, København V, Denmark) with 200 µL of complete Dulbecco’s modified Eagle medium (containing 10% fetal bovine serum (Gibco; Thermo Fisher Scientific, Waltham, MA, USA) and 1% penicillin–streptomycin (Gibco; Thermo Fisher Scientific, Waltham, MA, USA) and incubated for 1–2 d. Rotavirus Wa strain (G1P [8]) (Rotavirus A; ATCC CRL-2378.1, Manassas, VA, USA) was thawed from −80 °C storage and activated via incubation with trypsin (final concentration of 10 µg/mL) for 1 h at 37 °C. Serum samples and controls were serially diluted in duplicate in a 96-well U-bottom plate, followed by the addition of 80 µL of diluted rotavirus (536 FFU/80 µL in serum-free media) to each well. After 1 h of incubation at 37 °C, the mixture was transferred to washed MA104 cells and incubated at 37 °C for 20 h. Infected cells were fixed with 4.8% formaldehyde (Sigma, Taufkirchen, Germany), permeabilized with ice-cold methanol (Sigma, Taufkirchen, Germany), and blocked with 1% BSA and 0.5% Tween-20 in PBS. Sheep anti-rotavirus antibody (Thermo Fisher Scientific) (diluted 1:500) and rabbit anti-sheep IgG (H+L) HRP (Southern Biotech, Birmingham, AL, diluted 1:2000) were used for viral antigen detection. TrueBlue peroxidase substrate buffer (Seracare, Milford, MA, USA) was added, followed by plate washing and air drying. Foci counting was performed using AID vSpot (AID Autoimmun Diagnostika GmbH, Straßberg, Germany) or IRIS 2 (MABTECH, Nacka Strand, Sweden). Anti-rotavirus neutralizing titer in sera was defined as the dilution resulting in a 50% reduction in foci (FRNT50) compared with that in the rotavirus-only control, as analyzed using GraphPad Prism 10 software.

### 2.9. Statistical Analyses

Statistical differences between data groups were determined using GraphPad Prism 10 (GraphPad Software, Inc., San Diego, CA, USA) with the Mann–Whitney U test. Non-significant differences were indicated with *p*-values > 0.05, significant differences (*) were indicated with *p*-values < 0.05, and highly significant differences (**) were indicated with *p*-values < 0.01.

## 3. Results

### 3.1. Construction of Expression Vectors for the Soluble Expression of ENC Rotavirus VP8* Fusion Protein in E. coli

PNP-based vaccine platforms offer the potential to meet unmet medical needs for efficacy, safety, and stability. However, it is important to note that PNP-based vaccines commonly face challenges related to correct folding and soluble expression because of their complex multidomain structure.

**Figure 1:**
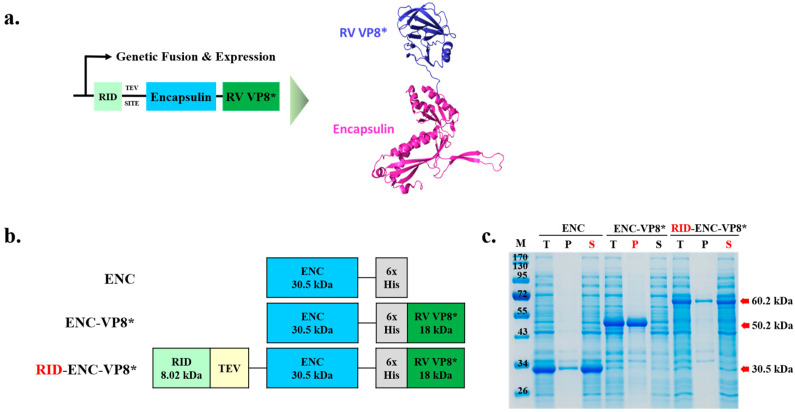
Design and soluble expression of the fusion protein of rotavirus receptor-binding domain and ENC nanoparticles. (**a**) Computational modeling and theoretical structure of ENC-RV VP8*. The predicted structure of ENC and linked VP8* are colored blue and green, respectively. The fusion protein of ENC-VP8* was predicted using SWISS-MODEL, Expasy. (**b**) Schematic designs of the protein structures. The size of proteins is shown under the name of each protein. (**c**) Expression of ENC and ENC-RV VP8* in the presence or absence of the RID fusion partner. The proteins were expressed at various vector constructions, and the cell lysates were separated into T, P, and S fractions via centrifugation and analyzed using 12% sodium dodecyl sulfate–polyacrylamide gel electrophoresis. ENC-VP8* with RID fusion partner is indicated by the red arrow. Lane M indicates molecular size markers. TEV SITE, tobacco etch virus (TEV) protease cleavage site; T, total fraction; P, insoluble pellet fraction; S, soluble fraction; ENC, encapsulin; RV, rotavirus vaccine, RID, RNA-interacting domain.

To address these challenges, we designed an expression strategy for efficient and soluble expression in bacteria and evaluated its enhanced immunogenicity (Figure 1a). Specifically, we fused the core region of the rotavirus VP8 protein (65–224 region fragment, 18 kDa) to the C-terminus of the ENC protein from T. maritima. Unlike ENC alone, the ENC-VP8* fusion protein (monomeric molecular weight 50.2 kDa) was initially expressed in a completely insoluble form in *E. coli* (Figure 1b).

Previous studies on protein folding have shown that RID-fused proteins exhibit increased solubility, prevent misfolding, and avoid the formation of nonfunctional aggregates [14,20]. Therefore, we positioned the RID at the N-terminus of the ENC-VP8* fusion protein to enhance its solubility and protein folding, thereby achieving high expression in *E. coli*. ENC (approximately 30.5 kDa), ENC-VP8 (approximately 50.2 kDa), and RID-ENC-VP8* (approximately 60.2 kDa) were expressed at the expected sizes (Figure 1c). Total cell lysates (T) were centrifuged and separated into pellet (P) and soluble (S) fractions. When expressed in *E coli* without RID tagging, ENC exhibited high solubility only in the absence of a C-terminal antigen. Consistent with previous studies [21,22], ENC spontaneously assembled into PNPs, and its structure was confirmed via size-exclusion chromatography (SEC) elution fractions using Superose™ 6 Increase 10/300 GL (Cytiva) (Figure S1b). While highly aggregated particles were predominantly observed in the 8–9 mL elution fraction, monomeric ENC PNPs were mainly eluted at 11–12 mL, as depicted in the SEC data (Figure S1a). Importantly, ENC-VP8* with the N-terminal RID tag exhibited a higher soluble fraction compared to ENC-VP8* alone (Figure 1c). These results demonstrate that the RID-tagged ENC presents potential as an NP platform, effectively increasing solubility and protein folding when fused with the target protein.

### 3.2. Enhanced Efficiency of TEV Cleavage and Assembly of ENC-VP8* via the Introduction of (GS)_3_ Linker

Reportedly, the resulting multidomain products often exhibit high instability despite each individual domain maintaining its structural and functional integrity [23]. Additionally, the introduction of an additional linker sequence could alleviate interference between domains [24]. Therefore, the linker sequence between the TEV cleavage site and ENC was introduced to enhance TEV cleavage efficiency with a 6×HIS tag positioned between the RID and TEV cleavage site to facilitate the removal of the N-terminal RID tag after the ENC-based protein is eluted using immobilized metal affinity chromatography (IMAC).

**Figure 2:**
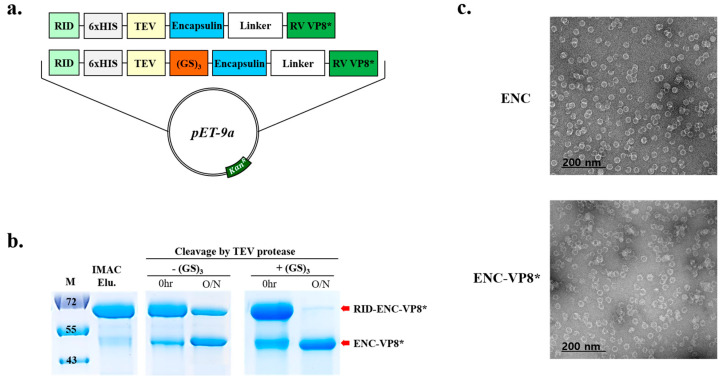
The addition of GS linker to the N-terminal of fusion protein increases the cleavage efficiency of TEV protease. (**a**) Schematic diagram of the expression vector system. The two linkers are shown in red (GS)_3_ and white (Linker) and TEV SITE indicates the TEV protease recognition site. (**b**) Time-lapse 12% sodium dodecyl sulfate–polyacrylamide gel electrophoresis analysis of TEV cleavage before (left panel) and after (right panel) the linker insertion. Lane M indicates molecular size markers. Proteins purified or separated until purification are indicated by red arrows. (**c**) Following IMAC elution, minor residual contaminants and non-target proteins were removed. Purified NPs (ENC NPs with and without VP8*) were negatively stained and analyzed via transmission electron microscopy. Scale bars = 100 nm and 200 nm. ENC, encapsulin; RV, rotavirus vaccine, RID, RNA-interacting domain; M, molecular weight marker; IMAC, elution with anion exchange chromatography.

Cleavage of RID from ENC-VP8* by TEV protease was insufficient even after overnight incubation with the protease (Figure 2b). Hence, we inserted an additional peptide linker (GS)_3_ between the TEV cleavage site and ENC to promote efficient TEV cleavage between the N-terminal RID tag (Figure 2a) and ENC until the fully cleaved ENC-VP8* product remained unmodified (Figure 2b, right panel). After optimization of TEV cleavage with the linker between the TEV cleavage site and the N-terminus of ENC, ENC-RV VP8* was purified. The introduction of an optimal length and suitable linker sequence between the TEV protease cleavage site and the N-terminus of ENC elicited a cleavage efficiency higher than 90%. Subsequently, the formation of ENC NPs displaying rotavirus VP8* on the surface after the removal of RID via treatment with TEV was confirmed via analysis using TEM (Figure 2c).

### 3.3. Role of L2 in Enhancing Solubility and High-Yield Expression of ENC-VP8* NPs

The α-helical forming of the linker (EAAAK)_n_ and highly random coiled conformation led by many (GGGGS)_n_ units control the distance and reduce the interference between the domains [16,17]. Numerous empirical studies have investigated the functionality of linkers in fusion proteins [25]. Linkers can enhance the folding and stability of fusion proteins, improving their expression yield and biological activity. Additionally, NPs can be linked to antigens, either through genetic fusion or chemical conjugation, leading to strongly enhanced immune responses via efficient tracking of particles to lymph nodes and activation of T- and B-lymphocytes [26].

**Figure 3:**
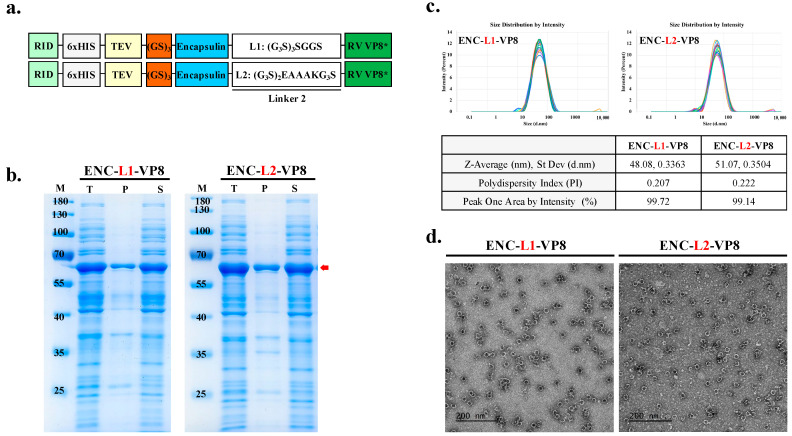
Linker affects the enhanced yield of nanoparticle (NP) antigen. (**a**) Schematic diagram of ENC-RV VP8* with inserted linker between ENC and VP8* antigen. Underlined L2 is colored as white. ENC and linked VP8* are colored blue and green, respectively. (**b**) The solubility of recombinant proteins fused with different linkers was analyzed with sodium dodecyl sulfate–polyacrylamide gel electrophoresis. Protein expression is under the control of isopropyl-β-D-thiogalactopyranoside induction. NP protein was fused with the C-terminal antigen VP8* via the linkers, L1: (G_3_S)_3_SGGS linker (left) and L2: (G_3_S)_2_EAAAKG_3_S linker (right). The arrow indicates the band position of each recombinant protein. (**c**) Dynamic light scattering measurement profiles (three independent scans of the same sample) of particle sizes and distributions of ENC NPs with L1 (left) and L2 (right). (**d**) Purified NPs were negatively stained and analyzed via transmission electron microscopy. Scale bar = 200 nm. M, molecular weight marker; T, total fraction; P, insoluble pellet fraction; S, soluble fraction; L1, linker 1; L2, linker 2; ENC, encapsulin; RV, rotavirus vaccine, RID, RNA-interacting domain.

To generate the vaccine candidate sequence, RV VP8* was linked using suitable linkers to connect the antigen to the ENC as an adjuvant to improve the immunogenicity of the vaccine construct. Previous studies [27,28] have highlighted the importance of appropriately lengthened linker sequences for thermal and pH stability. Furthermore, differences in amino acid sequences contribute to linker flexibility. Insertion of linkers between particles and epitopes is crucial for effective domain function and efficient separation [29]. Notably, linkers consisting of (G_4_S)n exhibit low antigenicity, whereas the EAAAK linker separates protein segments and adjuvant components to minimize interference. Thus, the amino acid sequences of the linkers between the particles and antigens were as follows: L1, flexible linker, (G_3_S)_3_SGGS; L2, linker with EAAAK, (G_3_S)_2_EAAAKG_3_S). The theoretical structure of the ENC-VP8* vaccine was modeled as the conjugation of the C-terminus of the 1–264 region of the structure of ENC from *T. maritima* with the N-terminus of the 65–224 region of the VP8 protein of RV VP8* through a pentapeptide linker sequence (G_3_S)_3_SGGS and (G_3_S)_2_EAAAK(G_3_S) to stabilize the formulation of C-terminal binding antigen (Figure 3a). Following the purification of soluble proteins (Figure 3b), we investigated the potential effects of the flexible linker L2, with EAAAK ([G_3_S]_2_EAAAKG_3_S) as a C-terminal protein conjugation linker for NP assembly.

As expected, ENC-VP8*, with each linker, assembled into well-formed NPs (50.2 kDa). The size of the purified ENC-VP8*NPs purified by the downstream process was further confirmed via DLS and TEM. The average NP diameter ranged from 40 to 50 nm (Figure 3c). TEM images of ENC-L1-VP8* and ENC-L2-VP8* structures revealed homogeneous hollow spherical particles (Figure 3d). Notably, DLS analysis indicated that ENC-L1-VP8* NP structures appeared slightly smaller, with an average intensity diameter of approximately 48 nm, compared with ENC-L2-VP8* NP, which had an average intensity distribution diameter of approximately 50 nm. Given that a signal was present for molecules approximately 50 nm in diameter, we concluded that there was the addition of the VP8* antigen, fused via a linker to the *T. maritima* ENC monomer, which had a size of 24 nm along with the N-terminal RID tags [30,31]. These findings align with previous studies suggesting that the L2 linker increases the distance between ENC particles and antigens by separating protein segments with the EAAAK alpha helix conformation. Interestingly, increased distance between the particles and antigens may further increase immunogenicity through stabilized particle formation. These results underscore the feasibility of establishing an ENC-based ENC-VP8* PNP manufacturing process sustainable for stable, high-yield candidate production. The purified ENC-VP8* PNPs meet the criteria for further investigation and appear sufficient to elicit a superior immune response compared to subunit vaccines that are currently under clinical study.

### 3.4. Simple Two-Step Purification Process Enables the Production of ENC-VP8* NPs in a Stable and Efficient Manner

NPs have the potential to serve as scaffolds for the delivery of biomolecules [32]. ENCs are thermostable and pH-stable components; therefore, they are extremely resistant to various stresses [31,33,34].

Herein, elementary buffer experiments using SDS-PAGE demonstrated that assembled ENC-VP8* was soluble at pH > 7.0 and over 90% at pH 7.5–11.0 (Figure S2a). A month-long stability study at pH 7.5 using TEM showed that the antigen-displaying ENC maintained its long-lasting stability (Figure S2b), and the purified ENC-VP8* NPs retained their stability over an extended period when stored at 4 °C (6 and 8 weeks).

### 3.5. Preparation of ENC-P2-VP8* and P2-VP8* for the Comparison Study

**Figure 4:**
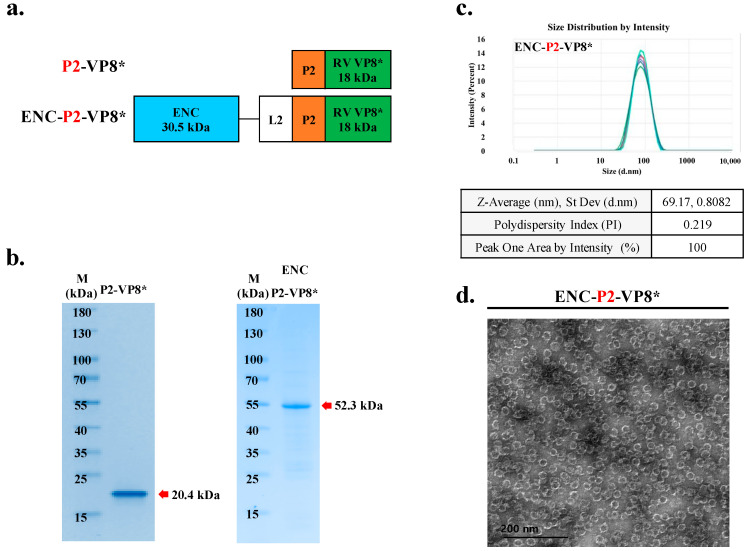
Conformation of VP8* displayed with P2 on ENC was self-assembled into 60-mer nanoparticles (NPs). (**a**) Schematic diagram of ENC-RV VP8* with inserted linker between ENC and VP8* antigen. P2 is colored orange. ENC and linked VP8* are colored blue and green, respectively. (**b**) Purified recombinant proteins fused with different linkers were analyzed with sodium dodecyl sulfate–polyacrylamide gel electrophoresis. NP protein was fused with the C-terminal antigen VP8* via the L2 linker with P2 (right). The arrow indicates the band position of each recombinant protein. (**c**) Dynamic light scattering measurement profiles (three independent scans of the same sample) of particle sizes and distributions of ENC NPs with P2. (**d**) Purified NPs were negatively stained and analyzed via transmission electron microscopy. Scale bar = 50 nm. ENC, encapsulin; L2, linker 2; RV, rotavirus vaccine; RSD, relative standard deviation.

Reportedly, the introduction of cluster of differentiation (CD)4+ universal T cell epitope P2, obtained from tetanus toxin on the N-terminus of VP8*, enhanced immunogenicity, which has been previously reported in a clinical study by PATH [18,35]. To achieve superior activity of PNP-based rotavirus over subunit vaccines, we established an expression construct in which the CD4 T cell epitope P2 was inserted between ENC-L2 and VP8* (Figure 4a). After purification using IMAC and IEX, it was found that P2-VP8* and ENC-P2-VP8* presented the expected molecular weights of 20.4 kDa and 52.3 kDa, respectively (Figure 4b).

To confirm their proper conformation and homogeneous assembly into ENC-P2-VP8* NPs, purified samples were further characterized using DLS (Figure 4c) and TEM (Figure 4d). DLS characterization indicated that purified ENC-P2-VP8* particles had a diameter of approximately 67 nm. The results of this study indicated that proteins expressed by bacteria with RID encoding ENC-P2-VP8* were perfectly folded and efficiently self-assembled into 60-mer NPs.

### 3.6. NP-Based ENC-P2-VP8* Enhances Binding and Neutralizing Antibody Responses Compared to the Subunit P2-VP8* in Mice

**Figure 5:**
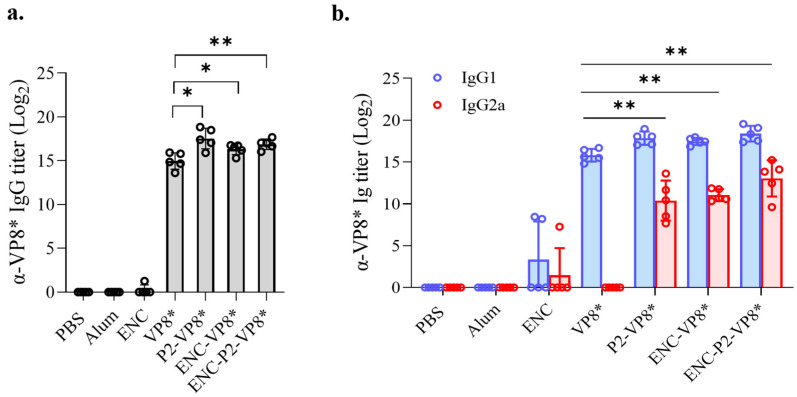
Comparison of IgG responses induced by VP8*, P2-VP8*, ENC-VP8*, and ENC-P2-VP8*. Mice were immunized with aluminum hydroxide-adjuvanted antigens thrice at 2-week intervals. VP8*-specific total IgG (**a**) and IgG1 and IgG2a (**b**) titers in serum were measured using enzyme-linked immunosorbent assay after three doses of 0.24 nmol of each antigen. Titers were expressed as reciprocal log_2_ of the serum dilution showing an optical density of 0.2 at 450 nm. The statistical marks are only expressed among groups administered with the VP8* series. The statistical bars between groups in (**b**) indicate statistical significance for both IgG1 and IgG2a in each group. *, *p* < 0.05; **, *p* < 0.01. PBS, phosphate-buffered saline; ENC, encapsulin.

Initially, VP8*-induced IgG responses were evaluated in the serum of the mice immunized with VP8*, P2-VP8*, ENC-VP8*, and ENC-P2-VP8*. After the third immunization with 0.24 nmol of each antigen formulated with aluminum hydroxide (Al[OH]_3_), groups that received P2-VP8*, ENC-VP8*, and ENC-P2-VP8* exhibited significantly higher levels of serum anti-VP8* IgG, IgG1, and IgG2a compared with those in the VP8* group (VP8* versus P2-VP8* and ENC-VP8*, *p* < 0.05; VP8* versus ENC-P2-VP8*, *p* < 0.01 for total IgG; and VP8* versus P2-VP8*, ENC-VP8*, and ENC-P2-VP8*, *p* < 0.01 for IgG1 and IgG2a) (Figure 5). This indicated that the fusion of either P2 epitope or ENC significantly enhances the Th1/Th2 balance of the core antigen VP8*, even in the presence of Al(OH)_3_, which is known as a Th2-skewing adjuvant [36]. However, it was not evident whether ENC and P2 synergistically enhanced antibody responses under the experimental conditions, as was observed when comparing the ENC-VP8* and ENC-P2-VP8* groups.

**Figure 6:**
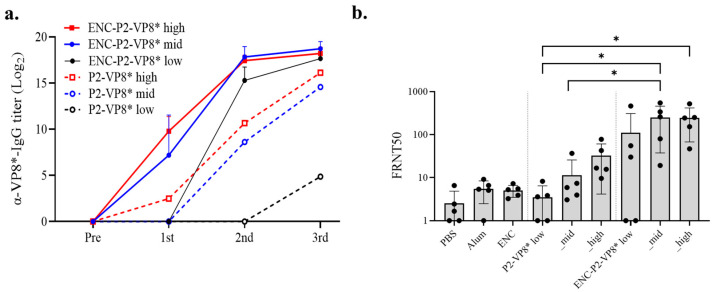
Detailed comparison of immune responses induced by P2-VP8* and ENC-P2-VP8*. (**a**) VP8*-specific total IgG titers at different doses and dosages (low, 0.01 nmol; mid, 0.05 nmol; high, 0.25 nmol) were measured using enzyme-linked immunosorbent assay. Results for the negative control groups (PBS, Alum, and ENC) are not shown. (**b**) Neutralizing antibody responses after three doses of the high dosage (0.25 nmol) were tested via FRNT. The FRNT50 of each serum was normalized via the quality control included in each plate. The Mann–Whitney test was used as statistical analysis and expressed only in P2-VP8* and ENC-P2-VP8* immunization groups. *, *p* < 0.05. PBS, phosphate-buffered saline; ENC, encapsulin.

Given the known superior immunogenicity of P2-VP8* compared to VP8* [37], we focused on comparing the immunogenicities of P2-VP8* and ENC-P2-VP8*. Immune responses at 0.01, 0.05, and 0.25 nmol of these two antigens were examined, referred to as low, mid, and high, respectively, with Al (OH)_3_ adjuvant. After the first immunization, the ENC-P2-VP8* high group exhibited a significant increase in VP8*-specific IgG titers compared with that in all P2-VP8* groups (low, mid, and high) and the ENC-P2-VP8* low group (*p* < 0.01). The ENC-P2-VP8* mid group showed significantly higher IgG levels after first immunization than the P2-VP8* mid and low groups and the ENC-P2-VP8* low group (*p* < 0.05). Following the second immunization, the ENC-P2-VP8* low group exhibited a significantly increased IgG titer compared with that in the P2-VP8* high and mid groups (*p* < 0.05) and the low group (*p* < 0.01). After the third immunization, an overall increase in IgG titer was observed in the P2-VP8* low group (Figure 6a). However, the ENC-P2-VP8* high and mid dose groups exhibited significantly higher IgG titers than all P2-VP8 dose groups (*p* < 0.05, *p* < 0.01, respectively). Additionally, the ENC-P2-VP8 low group exhibited significantly higher IgG levels than the P2-VP8* low group (*p* < 0.01) (Figure 6a and Table 1). This indicates that the NP structure, formed by the fusion of ENC to P2-VP8*, induced faster and more robust IgG responses than subunit P2-VP8*.

Neutralizing antibody responses against the homotypic P [8] rotavirus Wa strain were evaluated via FRNT with mouse immune sera. After the third immunization, the ENC-P2-VP8* low group presented a higher mean FRNT50 value than the P2-VP8* high group, although this was not a statistically significant difference. The ENC-P2-VP8* high and mid groups exhibited statistically significant increases compared with the P2-VP8* low group (*p* < 0.05). Furthermore, the ENC-P2-VP8* mid group also showed a significant increase in neutralizing antibody responses compared to the equimolar P2-VP8* mid group (*p* < 0.05) (Figure 6b). Despite the lack of statistical significance, the mean FRNT50 values were higher in the ENC-P2-VP8* low and high groups compared to the equimolar P2-VP8* groups. These findings suggest that the NP-based ENC-P2-VP8* formulation is more effective at inducing neutralizing antibodies against rotavirus than the subunit P2-VP8*.

## 4. Discussion

Design of antigen presentation ensuring high immunogenicity is crucial for vaccine efficacy and poses a fundamental challenge in vaccine development [38,39,40]. The assembly and display of antigens, such as virus-like particles (VLPs) and NPs, present a promising strategy because of their stability, biocompatibility, and ability to elicit robust immune responses via efficient delivery into antigen-presenting cells [41,42]. VLP/NPs display antigens on the surface of particles at high density and efficiently cross-link B-cell receptors, stimulating strong antibody responses to facilitate B and T cell interactions [43]. These attributes make particle-based platforms attractive for developing effective vaccines.

Protein nanoparticles are advantageous for drug and macromolecule delivery due to their biocompatibility, controlled release capabilities, targeted delivery potential, and high encapsulation efficiency [4,5]. They offer a versatile and customizable system that can be engineered for specific therapeutic needs. Bacterial proteins were chosen over denatured proteins, such as gelatin because they maintain structural integrity, allow for consistent and reproducible production, and can be easily modified for functional purposes [44]. Gelatin, on the other hand, may suffer from variability and loss of functional properties due to denaturation, making bacterial proteins a more reliable choice for nanoparticle applications.

Various types of inorganic and organic NPs have been engineered to improve their pharmacological properties and serve as promising delivery systems [42,45]. Unlike subunit vaccines that are composed of soluble monomeric antigens, assembling antigens in a multimeric and highly repetitive manner promotes robust humoral and cellular immune responses, making them superior vaccine design platforms [46]. For instance, human papillomavirus (HPV) vaccines based on HPV L1 VLP induce potent and long-lasting antibody responses, ensuring safety and high immunogenicity in humans [47,48,49]. Additionally, the need for high levels of neutralizing antibodies against severe acute respiratory syndrome coronavirus 2 favors recombinant proteins and NPs owing to their strong immunogenicity, safety profiles, and ability to increase neutralizing antibody titers [38,50]. In addition to protein-only assembled VLPs, such as HPV or hepatitis E virus, bacterial membrane vesicles offer alternative methods for displaying immunogens, including pathogen-associated molecular patterns, offering promising avenues for vaccine development against various pathogens [51].

Despite their advantages, challenges remain in the display and assembly of antigens, such as VLP/NP proteins in bacterial hosts. The chimerization of antigens with multimeric scaffolds can result in misfolded insoluble aggregates owing to the kinetic complexities in the folding pathway within the bacterial cytoplasm. This often necessitates in vitro refolding in the presence of high concentrations of chaotropic agents, compromising the immunological quality of antigens. To address this issue inherent to single-component chimeric approaches, a two-component system such as a SpyTag:SpyCatcher conjugation system could be employed [52,53].

In the context of rotavirus, the rotavirus VP8* core antigen, a proteolytic cleavage product of the outer capsid spike protein VP4, containing neutralizing epitopes, was fused with the universal CD4 T cell epitope P2 and expressed in *E. coli.* This development at National Institutes of Health (NIH) led to a trivalent formulation comprising genotypes P [8], P [6], and P [4,18,54], advanced clinically by PATH [19,37,55,56,57]. Furthermore, displaying the VP8* antigen on NPs has been an advanced approach to elicit strong anti-VP8* IgG antibody responses and high titers of functional antibodies tailored for low- and middle-income countries with high diarrheal mortality rates [7,58,59,60,61,62,63].

By utilizing innovative strategies of the RNA-mediated chaperone system, employing a RID fusion tag for tRNA interaction, we enhanced protein solubility by mimicking molecular chaperones. This approach prevents aggregation through hydrophobic shielding and electrostatic repulsion while ensuring proper folding kinetics [15], thus improving the solubility and folding of chimeric proteins and further increasing vaccine antigen production efficiency. To enhance efficacy and safety, we genetically engineered VP8* to display as a rotavirus-specific receptor-binding domain on the surface of a PNP carrier, demonstrating specific immunogenicity against authentic rotavirus strains. Thus, a tripartite modular design, RID-ENC-VP8*-RID, with the tRNA-binding tag as the folding catalyst, ENC as the scaffold for NP assembly, and VP8* as the target antigen, enabled expression of the chimeric protein predominantly in soluble form, self-assembled into NPs of the expected size.

ENC, a heat-stable protein cage NP isolated from *T. maritima*, assembles from 60 copies of identical 31 kDa monomers, forming a thin, icosahedral symmetric cage structure with interior and exterior diameters of 20 nm and 24 nm, respectively [33,64]. Their ability to self-assemble into stable NPs and accommodate various antigenic proteins makes them versatile platforms for vaccine development [52,65,66,67,68]. The results of this study further indicate that harnessing a novel RNA-mediated chaperone system allows proper folding of the antigen of interest for assembly in a bacterial host, enabling high-yield and large-scale production. Our findings, along with those of others, indicate that displaying antigens on NP-based platforms efficiently induces antibodies against the displayed antigens [60,69]. Furthermore, herein, the vaccine candidate showed stability at neutral pH for at least 8 weeks at temperatures exceeding standard room temperature. The findings of this study showed that purified NPs remained stable over extended periods, evidenced by durability over 6- and 8-week intervals (Figure S2b). The stability of the vaccine candidate under varying conditions in the experimental setting would benefit formulations, ensuring long-term storage and transportation.

Herein, we confirmed the significant enhancement of humoral immunogenicity, both in VP8*-specific IgG and neutralizing antibodies, by presenting the P2-VP8* antigen in a high-density, well-ordered array on NP surfaces. It is known that antigens displayed on NP surfaces induce more potent cell-mediated as well as humoral immune responses [45]. 

We extended the ENC-VP8* particle by fusing it with the universal CD4+ T cell epitope, P2, to enhance its immunogenicity [37]. Our initial assessment revealed that fusing either P2 or ENC significantly enhanced VP8*-specific IgG responses, including IgG1 and IgG2a, compared to VP8* alone. Notably, P2, ENC, or ENC-P2 fusion induced effective IgG2a responses, even in the presence of Al(OH)_3_, an adjuvant known to skew immune responses toward Th2 cells [36]. The detailed comparison showed that NP-based ENC-P2-VP8* significantly improved both IgG and neutralizing antibody responses across various dosages compared with subunit P2-VP8*. This enhancement in immunogenicity was likely due to increased antigen stability and presentation provided by the NP structure. The substantial increase in both IgG1 and IgG2 titers indicates that NP formulation promoted a balanced Th1/Th2 response, crucial for effective vaccination against viral infections. Furthermore, enhanced neutralizing antibody responses highlight the potential of ENC-P2-VP8* as a superior vaccine candidate compared to subunit P2-VP8*. These findings are consistent with those of previous studies demonstrating that NP-based vaccines can enhance immunogenicity by improving antigen delivery and presentation to the immune system [70].

In addition, Roier et al. reported that mRNA-based Lumazine synthase-P2-VP8* nanoparticle vaccines are highly immunogenic in rodents and benefit from the adjuvant effects of mRNA and lipid nanoparticles [58]. However, in addition to ensuring the safety of newborns, considering the main vaccination target is infants in low-income countries, issues regarding storage and transportation will be the challenging obstacles to overcome. Our ENC-P2-VP8* nanoparticle vaccine, produced in E. coli, offers notable advantages in terms of safety, stability, and cost-effectiveness. The demonstrated safety of ENC-P2-VP8* in mice, along with its stable formulation and well-characterized production process, makes it a promising candidate for safe and effective widespread vaccination in young populations.

Rotavirus infection is known to have a viremic phase [71,72], necessitating effective immune responses providing backup protection by targeting infected cells beyond mucosal surfaces. While mucosal immunity is critical for preventing initial infection and controlling rotavirus replication at mucosal surfaces, systemic cell-mediated immunity becomes crucial for combating systemic spread of infection and clearing infected cells throughout the body [73]. In the context of enteric infections, the induction of systemic cell-mediated immunity provides an additional layer of defense against gastrointestinal pathogens, particularly when mucosal immunity alone may not suffice to prevent infection [74].

Thus, further investigation is required to determine whether the improved humoral and cellular immunity induced by ENC-P2-VP8* administration can lead to enhanced protective immunity against rotavirus infection and resultant symptoms in appropriate animal models in future studies. Following modest immune responses observed in mouse models [18,75] and more robust responses in guinea pigs [35,54,57], further studies in gnotobiotic pig models are needed to evaluate ENC-P2-VP8* efficacy against RV infection.

This exploratory study of ENC-based NP platforms harnessed with a novel RNA-based chaperone system establishes a robust and versatile platform for recombinant vaccines against viral infections.

## 5. Conclusions

In conclusion, the findings of this study support the development of a versatile vaccine consisting of an ENC displaying the rotavirus antigen VP8*. This approach has been shown to present antigens on self-assembled NPs and enhances immunogenicity by eliciting VP8*-specific IgG and neutralizing antibodies, thus offering a promising alternative to rotavirus vaccination. Additionally, robust bacterial production may enable the fast delivery of innovative vaccines and mitigate the threats to global health.

## 6. Patents

D. C. and H. J. of InThera, Inc. have registered a patent filing to encapsulate the target protein-RID platform (KR10-2264535), “Recombinant Expression Vector for Production Of Encapsulin-Based Vaccine and Method for Manufacturing the Same” related to the PCT application (PCT/KR2021/006758).

## Figures and Tables

**Table 1:** Statistical results for VP8*-specific IgG titers in Figure 6a based on the number of doses and the dosage amounts of P2-VP8* and encapsulin-P2-VP8*.

Antigen	Dose	ENC-P2-VP8* Mid	ENC-P2-VP8* Low	P2-VP8* High	P2-VP8* Mid	P2-VP8* Low
ENC-P2-VP8* high	1st	ns (*p* = 0.6905)	** (*p* = 0.0079)	** (*p* = 0.0079)	** (*p* = 0.0079)	** (*p* = 0.0079)
	2nd	ns (*p* = 0.4206)	* (*p* = 0.0317)	** (*p* = 0.0079)	** (*p* = 0.0079)	** (*p* = 0.0079)
	3rd	ns (*p* = 0.4206)	ns (*p* = 0.2222)	* (*p* = 0.0159)	* (*p* = 0.0159)	** (*p* = 0.0079)
ENC-P2-VP8* mid	1st	-	* (*p* = 0.0476)	ns (*p* = 0.1032)	* (*p* = 0.0476)	* (*p* = 0.0476)
	2nd	-	* (*p* = 0.0159)	** (*p* = 0.0079)	** (*p* = 0.0079)	** (*p* = 0.0079)
	3rd	-	ns (*p* = 0.0952)	** (*p* = 0.0079)	** (*p* = 0.0079)	** (*p* = 0.0079)
ENC-P2-VP8* low	1st	-	-	ns (*p* = 0.4444)	ns (*p* > 0.9999)	ns (*p* > 0.9999)
	2nd	-	-	* (*p* = 0.0159)	* (*p* = 0.0159)	** (*p* = 0.0079)
	3rd	-	-	ns (*p* = 0.0556)	ns (*p* = 0.0556)	** (*p* = 0.0079)
P2-VP8* high	1st	-	-	-	ns (*p* = 0.4444)	ns (*p* = 0.4444)
	2nd	-	-	-	ns (*p* = 0.1508)	** (*p* = 0.0079)
	3rd	-	-	-	ns (*p* = 0.2222)	** (*p* = 0.0079)
P2-VP8* mid	1st	-	-	-	-	ns (*p* > 0.9999)
	2nd	-	-	-	-	** (*p* = 0.0079)
	3rd	-	-	-	-	** (*p* = 0.0079)

ENC, encapsulin; ns, not significant; *, *p* < 0.05; **, *p* < 0.01. High: 0.25 nmol; mid: 0.05 nmol; and low: 0.01 nmol. Mann–Whitney test was used for statistical analysis.

## Data Availability

The resources and materials used in the manuscript are available from the corresponding author upon request. Any such requests should be directed to and will be fulfilled by Deog-Young Choi (dychoi@inthera.co.kr) or Jae Ouk Kim (jokim@ivi.int).

## Supplementary Materials

The following supporting information can be downloaded at: https://www.mdpi.com/article/10.3390/vaccines12091020/s1, Figure S1. Characterization of encapsulin nanopoarticles (NPs) with size exclusion chromatography (SEC). Figure S2. Stability of NP particles in adjusted pH maintains its formation of particle. Figure S3. Protein purification of P2-VP8* in the *E. coli* with RID fusion partner.
