# Supplementary material for: Molecular Design of Encapsulin Protein Nanoparticles to Display Rotavirus Antigens for Enhancing Immunogenicity
Source: Vaccines (Basel). 2024 Sep 6;12(9):1020. doi: 10.3390/vaccines12091020 (PMC11435836; doi:10.3390/vaccines12091020)
Supplement: Supplementary file 1 [file vaccines-12-01020-s001.zip › Raw Data for DLS Measurements_vaccines-3164257.pptx]

## Slide 1
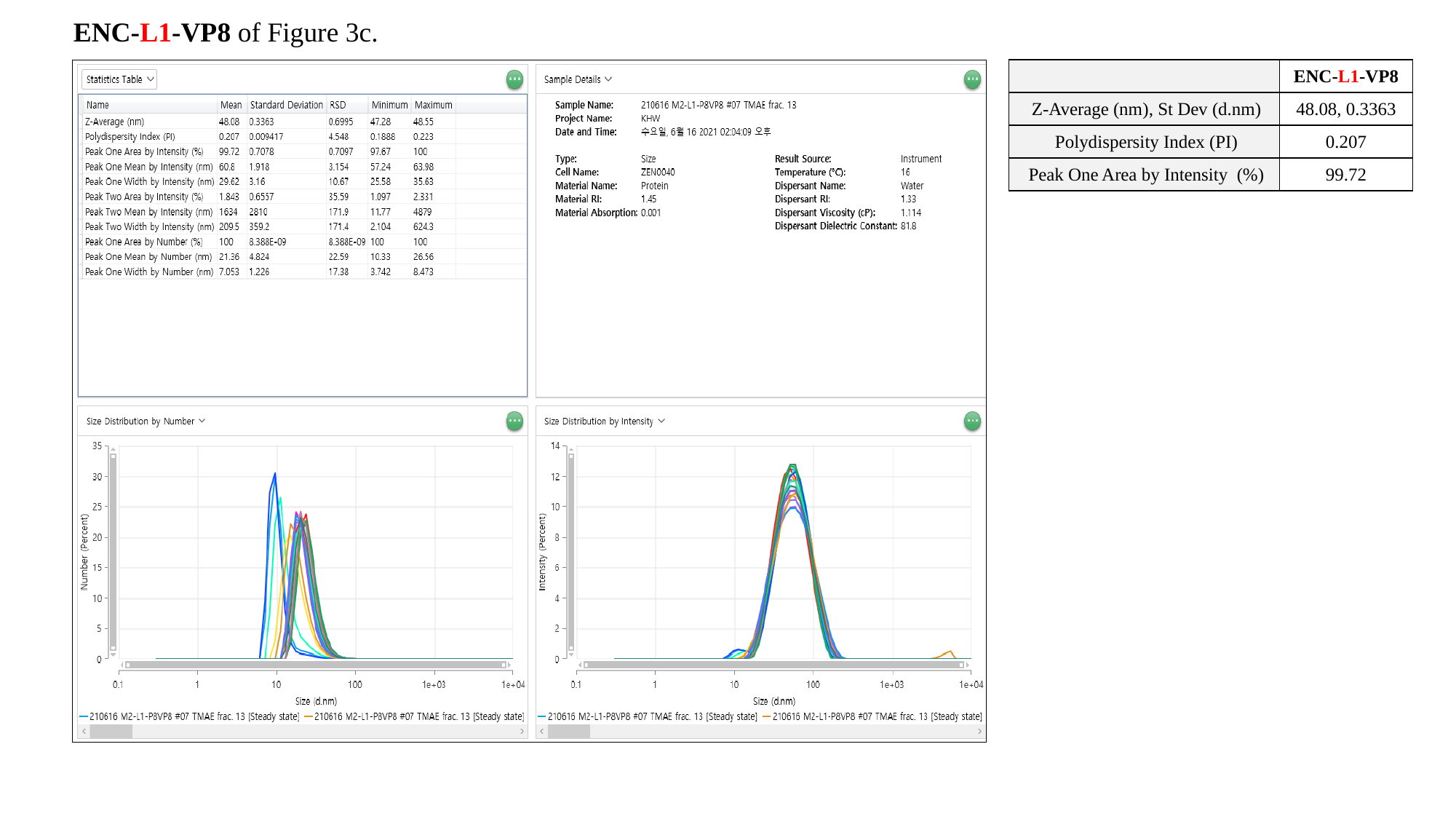

ENC-L1-VP8 of Figure 3c.
| | ENC-L1-VP8 |
| --- | --- |
| Z-Average (nm)​, St Dev (d.nm) | 48.08, 0.3363 |
| Polydispersity Index (PI)​ | 0.207 |
| Peak One Area by Intensity  (%)​ | 99.72 |

## Slide 2
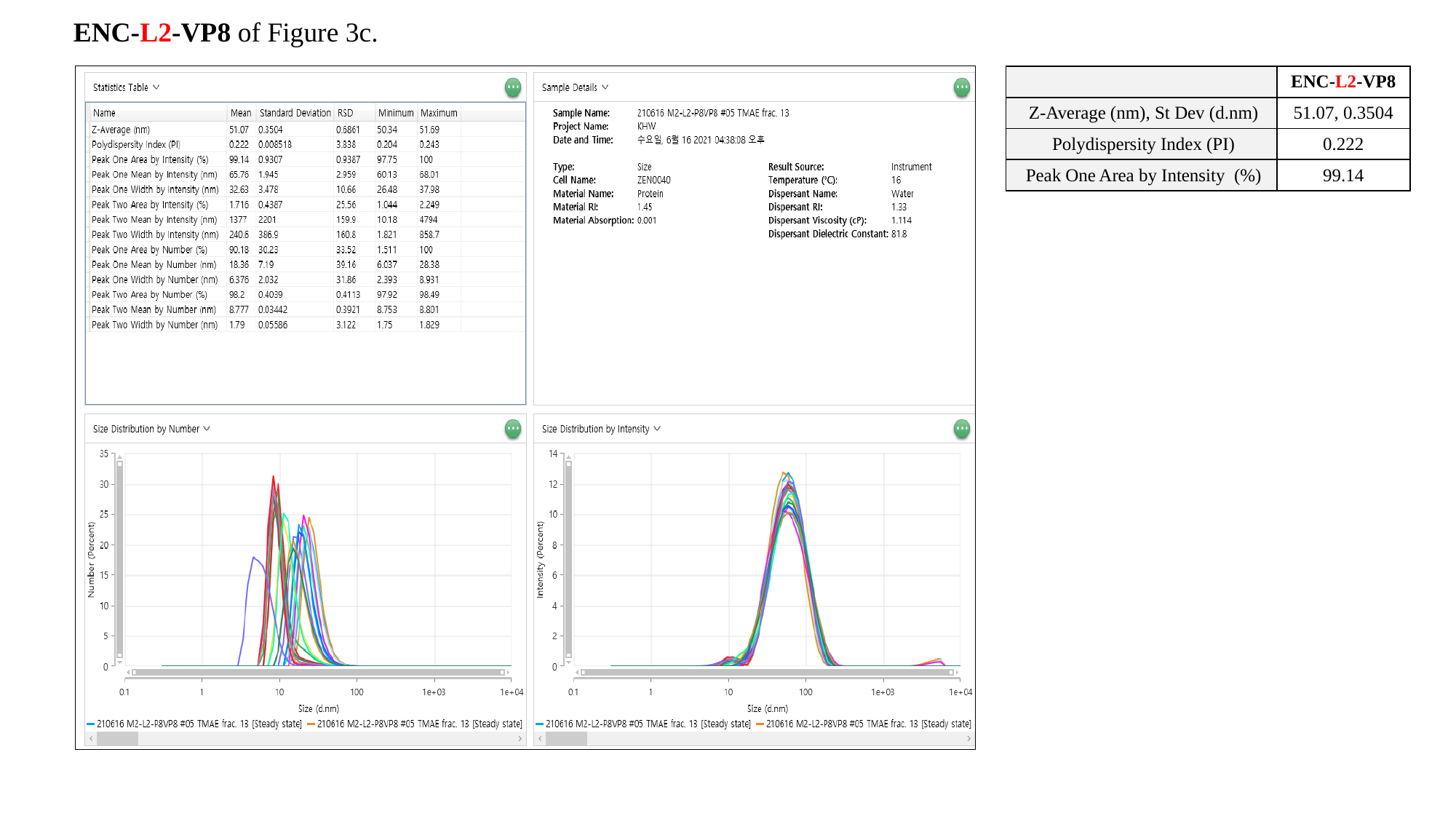

ENC-L2-VP8 of Figure 3c.
| | ENC-L2-VP8 |
| --- | --- |
| Z-Average (nm)​, St Dev (d.nm) | 51.07, 0.3504 |
| Polydispersity Index (PI)​ | 0.222 |
| Peak One Area by Intensity  (%)​ | 99.14 |

## Slide 3
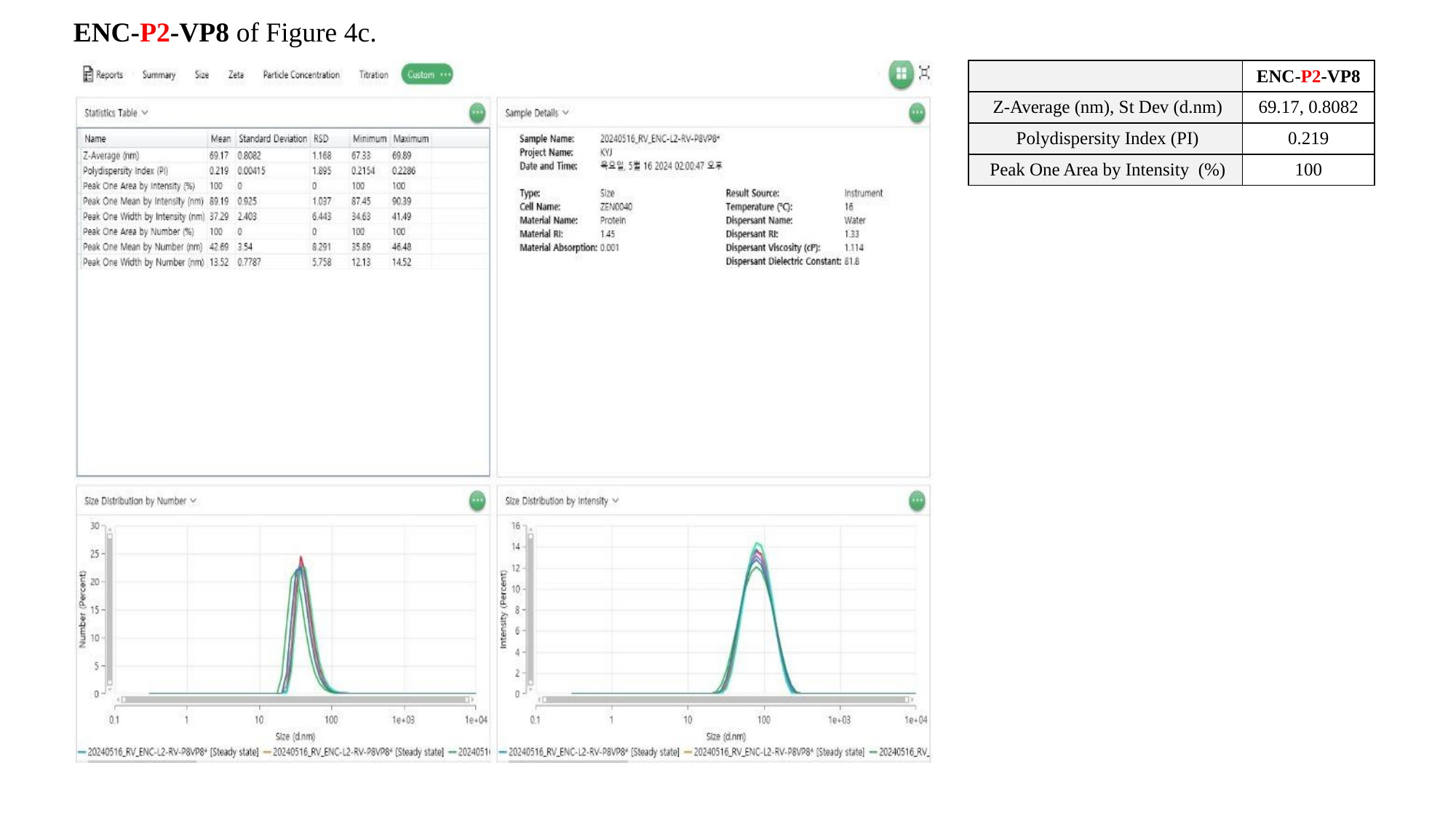

ENC-P2-VP8 of Figure 4c.
| | ENC-P2-VP8 |
| --- | --- |
| Z-Average (nm)​, St Dev (d.nm) | 69.17, 0.8082 |
| Polydispersity Index (PI)​ | 0.219 |
| Peak One Area by Intensity  (%)​ | 100 |
