# Supplementary material for: Molecular Design of Encapsulin Protein Nanoparticles to Display Rotavirus Antigens for Enhancing Immunogenicity
Source: Vaccines (Basel). 2024 Sep 6;12(9):1020. doi: 10.3390/vaccines12091020 (PMC11435836; doi:10.3390/vaccines12091020)
Supplement: Supplementary file 1 [file vaccines-12-01020-s001.zip › vaccines-3164257-supplementary.pdf]

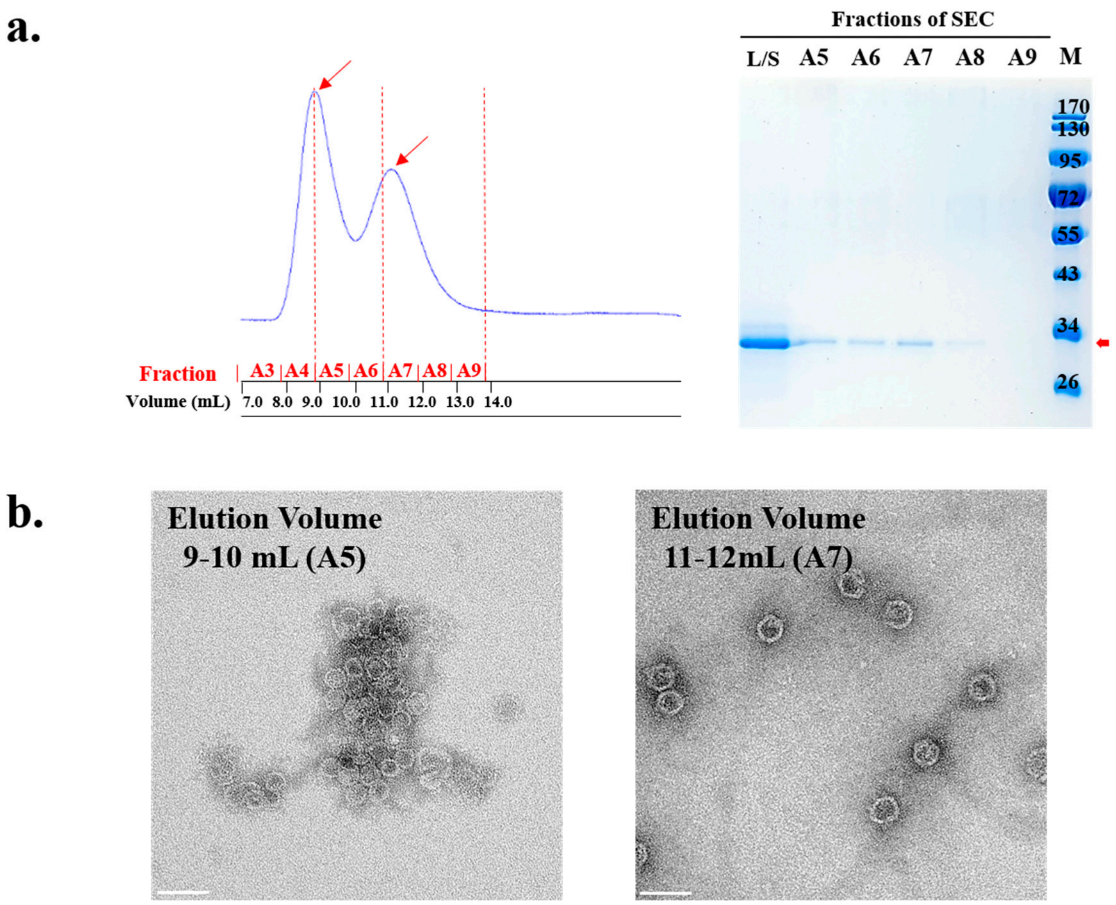

Supplementary Figure S1. Characterization of Encapsulin Nanopoarticles (NPs) with Size exclusion chromatography (SEC). (**a**) Proteins purified or separated until purification were indicated by red arrows. SEC for the analysis was conducted as the SEC profile (Left panel). Protein of size exclusion chromatography of ENC were analyzed by SDS-PAGE (Right panel). Proteins purified or separated until purification were indicated by red arrows. M: molecular weight marker; :L/S: loading sample; A5~A9: eluted fractions with size exclusion chromatography. Proteins purified or separated until purification were indicated by red arrows. (**b**) After size exclusion chromatography, Purified ENC NPs of Elution volume 9-10 mL (Left) and 11-12 mL (Right) were negatively stained and analyzed by Transmission Electron Microscopy. Scale bars = 50 nm.

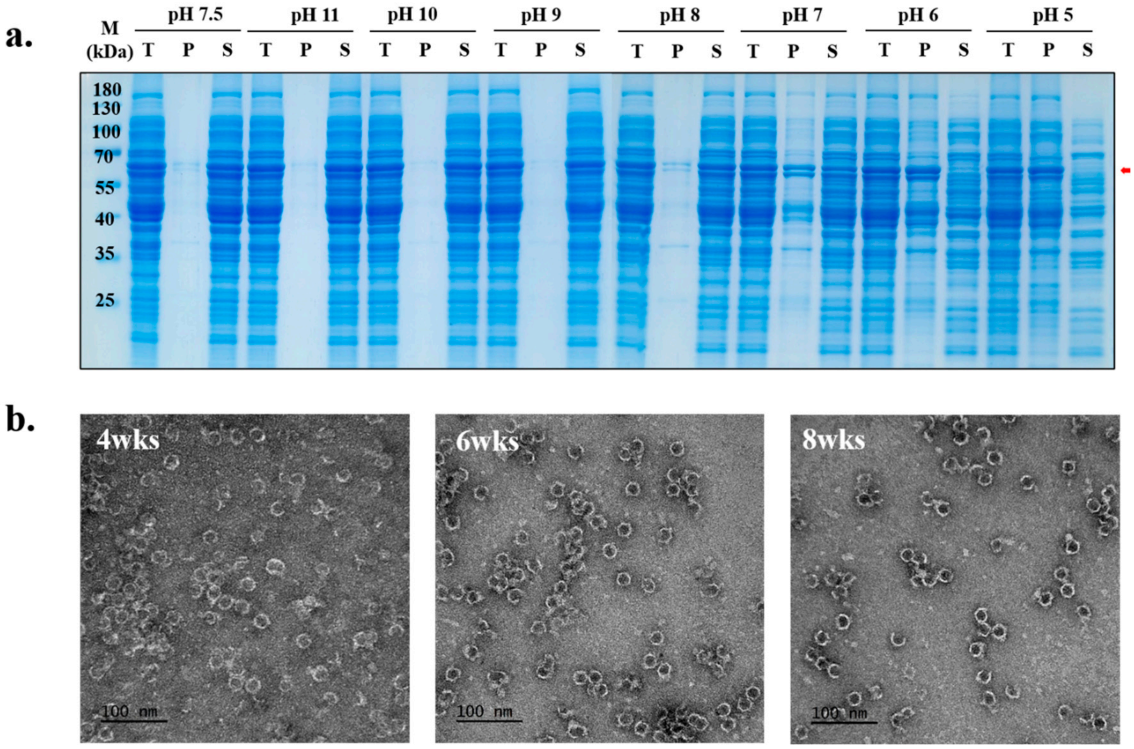

Supplementary Figure S2. Stability of NP particles in adjusted pH maintains its formation of particle. (**a**) Effect of pH on the protein solublity of ENC-VP8 NP in different values pH5.0 to pH11.0. Proteins that aggregate under stress conditions are generally acidic. (**b**) Stability of Purified NPs in optimized pH were negatively stained and analyzed by Transmission Electron Microscopy. Scale bar = 100 nm.

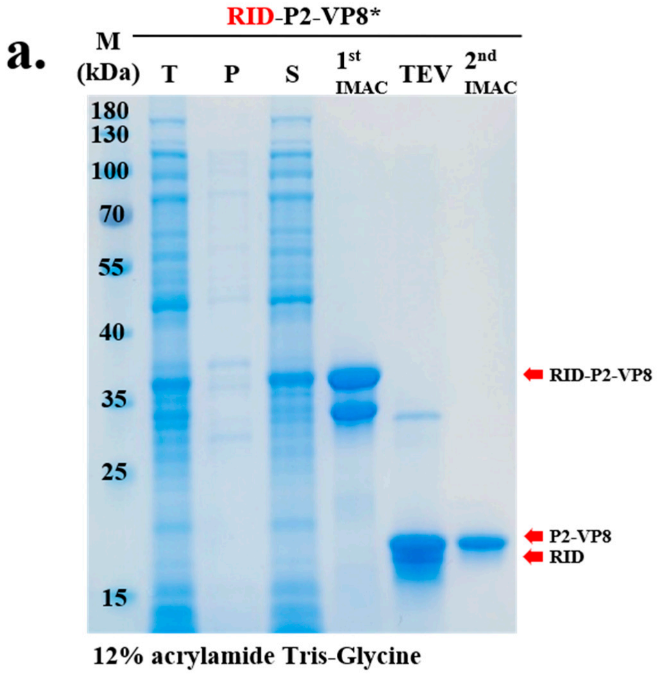

Supplementary Figure S3. Protein Purification of P2-VP8* in the e.coli with RID fusion partner. (**a**) Expression P2-RV VP8* in the e.coli with RID fusion partner. The cell lysate was separated into total (T), insoluble pellet (P), Soluble (S) fractions by centrifugation. SDS-PAGE analysis following purification profile of P2-VP8* with N-terminal tagged RID purified by two-step elution. M: molecular weight marker; IMAC: purified with anion exchange chromatography; TEV: cleaved with tev protease.
